# Epigenetic Modifications Are Involved in Transgenerational Inheritance of Cadmium Reproductive Toxicity in Mouse Oocytes

**DOI:** 10.3390/ijms252010996

**Published:** 2024-10-12

**Authors:** Jiaqiao Zhu, Shuai Guo, Jiangqin Cao, Hangbin Zhao, Yonggang Ma, Hui Zou, Huiming Ju, Zongping Liu, Junwei Li

**Affiliations:** 1 College of Veterinary Medicine, Yangzhou University, Yangzhou 225009, China; sguo@innosar.cn (S.G.); penguin_jq@163.com (J.C.); nuyoahzhb@163.com (H.Z.); 007854@yzu.edu.cn (Y.M.); zouhui@yzu.edu.cn (H.Z.); hmju@yzu.edu.cn (H.J.); liuzongping@yzu.edu.cn (Z.L.); 2 Guangling College, Yangzhou University, Yangzhou 225000, China; 3 Jiangsu Co-Innovation Center for Prevention and Control of Important Animal Infectious Diseases and Zoonoses, Yangzhou 225009, China; 4 Joint International Research Laboratory of Agriculture and Agri-Product Safety, The Ministry of Education of China, Yangzhou University, Yangzhou 225009, China

**Keywords:** cadmium, offspring oocytes, epigenetic modifications, oocyte maturation

## Abstract

Maternal cadmium exposure during pregnancy has been demonstrated to have detrimental effects on offspring development. However, the impact of maternal cadmium exposure on offspring oocytes remains largely unknown, and the underlying mechanisms are not fully understood. In this study, we found that maternal cadmium exposure during pregnancy resulted in selective alteration in epigenetic modifications of mouse oocytes in offspring, including a decrease in H3K4me2 and H4K12ac, as well as an increase in DNA methylation of *H19*. Although ROS levels and mitochondrial activity remain at normal levels, the DNA damage marker γH2AX was significantly increased and the DNA repair marker DNA-PKcs was remarkably decreased in offspring oocytes from maternal cadmium exposure. These alterations are responsible for the decrease in the quality of mouse oocytes in offspring induced by maternal cadmium exposure. As a result, the meiotic maturation of oocytes and subsequent early embryonic development are influenced by maternal cadmium exposure. RNA-seq results showed that maternal cadmium exposure elicits modifications in the expression of genes associated with metabolism, signal transduction, and endocrine regulation in offspring ovaries, which also contribute to the disorders of oocyte maturation and failures in early embryonic development. Our research provides direct evidence of transgenerational epigenetic inheritance of cadmium reproductive toxicity in mouse germ cells.

## 1. Introduction

Cadmium is an environmental pollutant that is widely present in soil, water, and air due to industrial activities. It easily enters the food chain and accumulates in various organs of humans due to its long biological half-life (10–35 years). Extensive research has demonstrated that cadmium exposure can lead to multiple forms of systemic toxicity, such as in skeletal, urinary, reproductive, cardiovascular, nervous, and respiratory systems [1,2]. The reproduction of both males and females is disrupted by cadmium even at low doses. For example, abnormal sperm development, decreased semen quality, menstrual cycle disorders, delay in puberty, hormone synthesis and release disorders, etc. [3,4]. We previously reported that maternal exposure to cadmium decreased oocyte quality by impairing meiotic maturation and impaired early preimplantation embryonic development by disturbing histone modifications and DNA methylation [5,6].

In addition to directly harming the female itself, cadmium exposure during pregnancy not only affects the maintenance of pregnancy but also has adverse effects on offspring development. There is a significant negative correlation between maternal cadmium levels and fetus development, including fetal growth restriction, low birth weight, and height [7,8,9]. Maternal cadmium exposure is also associated with deteriorations in cognitive functions and neurobehavioral function of offspring by affecting neural development [10,11]. Indeed, maternal exposure inevitably results in exposure of germ cells, which means direct exposure of offspring as well to some degree [12]. It has been reported that the developmental toxicity of cadmium on *Drosophila* and *Danio rerio* can be transmitted for two or three generations [13,14,15]. However, the impact of maternal cadmium exposure on offspring reproduction is not well understood. This would involve studying the reproductive health of the offspring and their ability to produce viable offspring themselves. Unlike spermatogenesis, which is a continuous process throughout the reproductive lifetime of males, early stages of oogenesis including the differentiation of oogonia are completed during the fetal period in females. This means that any defects or disruptions in oogonia caused by maternal cadmium exposure during pregnancy can potentially have long-lasting effects, which result in a decrease in the quality of offspring oocytes.

The toxic effects of cadmium are multifaceted and involve several specific mechanisms. Oxidative stress, DNA damage, inflammation, metabolism, endoplasmic reticulum stress, hormone signaling, apoptosis, autophagy, and epigenetic changes are among the key mechanisms implicated in cadmium toxicity [16,17,18]. Recent evidence suggests that maternal exposure to cadmium during pregnancy can lead to DNA hypomethylation in the blood of offspring from birth up to prepubertal age [19]. Epigenetic changes including DNA methylation and histone modification play a crucial role in regulating gene expression and cellular functions. Alterations in epigenetic marks can have profound effects on the phenotype of individuals and offspring. The germline transmission of epigenetic changes is required for transgenerational epigenetic inheritance, which is induced by environmental toxicants [20]. Thus, the exact mechanism of cadmium toxicity on offspring germ cells remains to be clarified. In the current paper, we aim to investigate the effects of maternal cadmium exposure during pregnancy on the oocytes of F1 mice and shed light on the underlying mechanisms.

## 2. Results

### 2.1. Cadmium Exposure during Pregnancy Reduced the Body Weight and the Ovary Coefficient of F1 Female Mice

**Figure 1:**
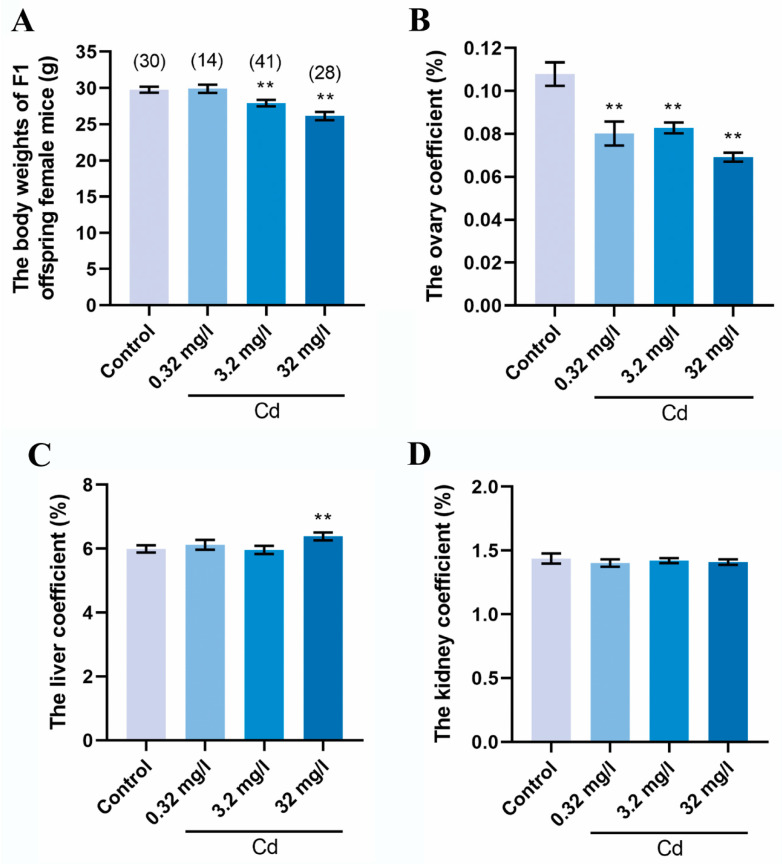
Cadmium exposure during pregnancy reduced the body weight and the ovary coefficient of F1 female mice. (**A**) The body weight of F1 female mice at 8 weeks old in each group. The total number of mice examined is given in parentheses at the top of each column. The organ coefficients of ovary (**B**), liver (**C**), and kidney (**D**) of F1 female mice. Data are presented as means ± the standard error of the mean (SEM). ** *p* values of <0.01.

We first measured the body weight and organ weight of an F1 female mouse. As shown in Figure 1A, the F1 female mouse from cadmium exposure during pregnancy showed a gradual decrease in body weight in a slightly dose-dependent manner. The body weight values of F1 female mice in the 3.2 and 32 mg/L Cd groups were significantly lower than that in the control group (control = 29.76 ± 0.40; 3.2 mg/L Cd = 27.92 ± 0.45; and 32 mg/L Cd = 26.14 ± 0.56; *p* < 0.01). The organ coefficients of ovary, liver, and kidney were calculated. As shown in Figure 1B, the ovary coefficient of F1 female mice in the groups of cadmium exposure was significantly lower than that in the control group (*p* < 0.01). The ovary coefficient in the control, 0.32, 3.2, and 32 mg/L Cd groups were 0.1079% ± 0.0055%, 0.0801% ± 0.0056%, 0.0828% ± 0.0025%, and 0.0692% ± 0.0021%, respectively. However, the liver coefficient showed comparable values between the control group and the cadmium exposure groups except for 32 mg/L (Figure 1C). There was no significant difference in the kidney coefficient between all groups (Figure 1D). Our results indicated that cadmium exposure during pregnancy affects the development of the body and ovaries of F1 female mice.

### 2.2. Cadmium Exposure during Pregnancy Reduced the Number of Ovulated Oocytes and Impaired the Oocyte Maturation in F1 Mice

**Figure 2:**
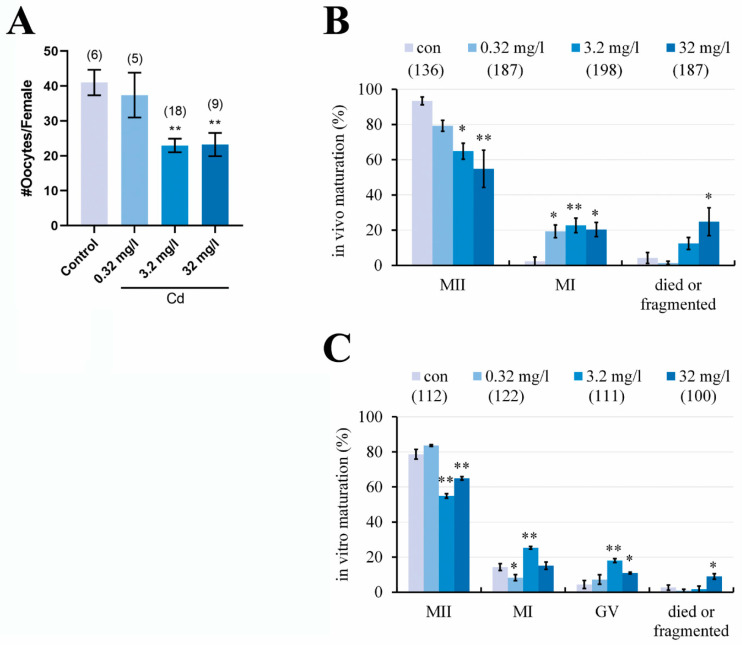
Cadmium exposure during pregnancy reduced the number of ovulated oocytes and impaired the oocyte maturation in vivo and in vitro. (**A**) The number of ovulated oocytes from F1 female mice after superovulation. (**B**) In vivo maturation rate of ovulated oocytes. (**C**) In vitro maturation rate of GV-oocytes. The total number of females (**A**) or oocytes (**B**,**C**) examined in each group is given in parentheses at the top of each column. *, ** *p* values of <0.05 and 0.01, respectively.

Although cadmium exposure during pregnancy affects ovary development, we asked whether cadmium exposure affects the ovulation of F1 female mice. As shown in Figure 2A, the number of ovulated oocytes in the 3.2 and 32 mg/L Cd groups was significantly less than that in the control group (control = 41.00 ± 3.66; 3.2 mg/L Cd = 22.94 ± 1.94; and 32 mg/L Cd = 23.22 ± 3.32/female; *p* < 0.01). The morphology of the ovulated oocytes from all groups was further examined under the stereo microscope. As shown in Figure 2B, although most oocytes in all groups were at the MII stage, which had extruded the first polar body, the percentages of MII-oocytes in the 3.2 and 32 mg/L Cd groups were significantly smaller than that in the control group (control = 93.39% ± 2.27%; 3.2 mg/L Cd = 64.85% ± 4.55%, *p* < 0.05; and 32 mg/L Cd = 54.84% ± 10.57%, *p* < 0.01). The effect of cadmium on the in vivo maturation of oocytes (MII-oocytes) shows dose dependence. Conversely, the percentage of MI-oocytes without the first polar body in the groups of cadmium exposure was significantly higher than that in the control group. These results indicated that cadmium exposure during pregnancy impairs the oocyte maturation in vivo.

Cadmium exposure may directly affect the oocyte maturation or indirectly affect it through the ovarian follicle microenvironment [19,21]. So, the GV-oocytes were isolated from the follicular environment and cultured in vitro for maturation. The results showed (Figure 2C) that the percentage of MII-oocytes was significantly smaller (*p* < 0.01) in the 3.2 and 32 mg/L Cd groups (54.87% ± 1.30% and 64.90% ± 0.97%, respectively) compared with the control group (78.65% ± 2.79%). Conversely, the percentages of MI-oocytes and GV-oocytes in the 3.2 and 32 mg/L Cd groups after in vitro maturation were significantly higher than those in the control group. These results indicated that cadmium exposure during pregnancy directly affects the oocyte maturation of F1 female mice.

### 2.3. Cadmium Exposure during Pregnancy Impairs the Embryonic Development in F1 Mice

**Figure 3:**
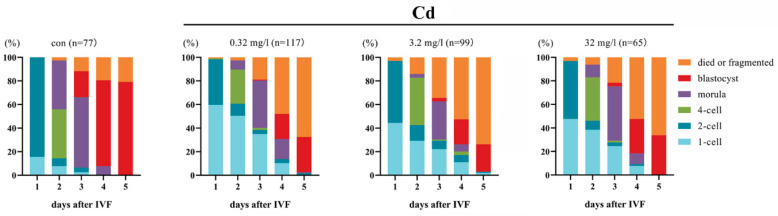
Cadmium exposure during pregnancy impairs the embryonic development of in vitro fertilized eggs (IVF) in F1 female mice. Each column indicates the percentages of embryos at different stages at 1–5 days in culture. The data were pooled from 4 experiments. The total number of embryos examined is given in parentheses at the top of each graph.

Most oocytes from cadmium exposure were at the MII stage, so the fertilization ability of oocytes and subsequent embryonic development were assessed by IVF and in vitro culture. The results are summarized in Figure 3. The rate of the first cleavage (2-cell embryo) from the groups of cadmium exposure was significantly lower (0.32 mg/L Cd = 38.46%; 3.2 mg/L Cd = 52.53%; and 32 mg/L Cd = 49.23%; *p* < 0.01) compared with the control group (84.42%). After culture for 5 days, a small proportion of embryos developed to the blastocyst stage in the groups of cadmium exposure (0.32 mg/L Cd = 29.92%; 3.2 mg/L Cd = 23.23%; and 32 mg/L Cd = 33.85%; *p* < 0.01), while 79.22% of embryos did so in the control group. More than 66% of embryos from cadmium exposure died or fragmented after 5 days, but the proportion in the control group was only 20.79%. Our results indicated that cadmium exposure during pregnancy leads to a decrease in the fertilization ability of F1 mouse oocytes and impairs subsequent embryonic development.

### 2.4. Cadmium Exposure during Pregnancy Did Not Affect Meiotic Spindle Morphology in F1 Mouse Oocytes

**Figure 4:**
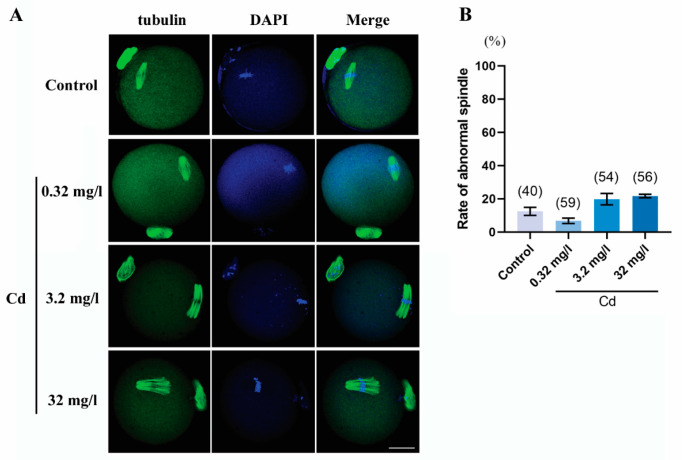
Cadmium exposure during pregnancy did not affect meiotic spindle morphology in F1 mouse oocytes. (**A**) Representative images of F1 mouse oocytes stained with anti-α-tubulin-FITC (green) and DAPI (blue). Scale bar, 20 μm. (**B**) The rate of abnormal spindle morphology. The total number of oocytes examined is given in parentheses at the top of each column.

The spindle morphology and the chromosome alignment of F1 mouse MII-oocytes were also assessed by immunofluorescence staining. As presented in Figure 4, the spindle was scattered and multipolar in the 3.2 and 32 mg/L Cd groups, but statistical results showed that there was no significant difference in abnormal spindle rate between the groups of cadmium exposure and the control group. However, the chromosomes were arranged in a lineup at the equatorial plane of the spindle in all groups, even if the spindle morphology was abnormal. These results indicated that cadmium exposure during pregnancy did not affect the maintenance of meiotic spindle morphology and the normal alignment of chromosomes.

### 2.5. Cadmium Exposure during Pregnancy Decreased H3K4me2 and H4K12ac in F1 Mouse Oocytes

**Figure 5:**
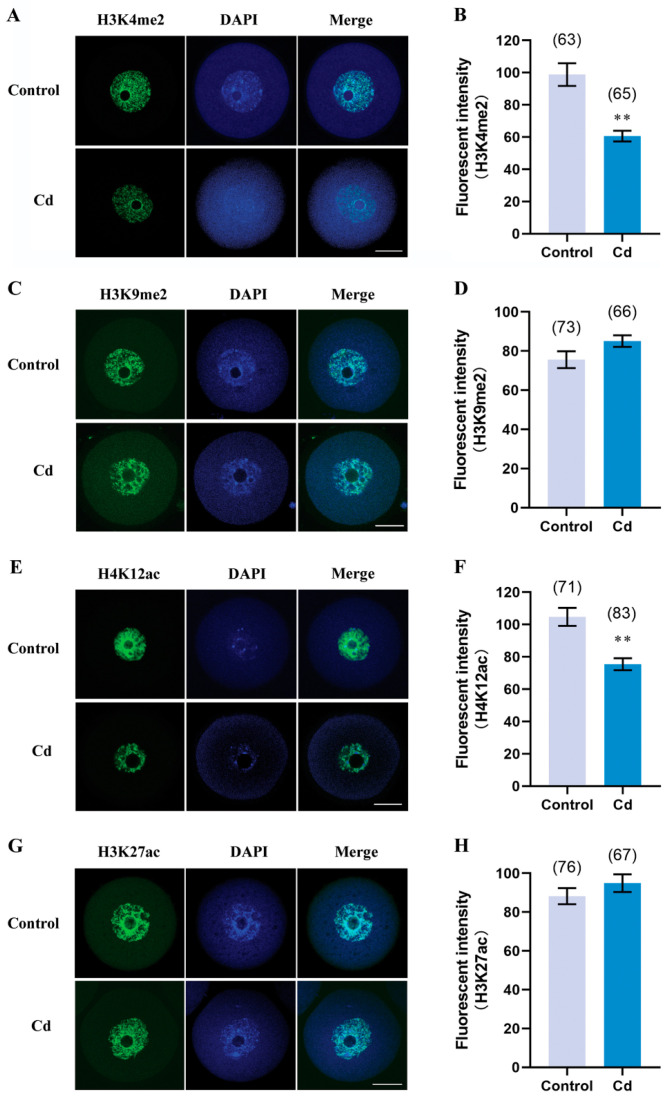
Cadmium exposure during pregnancy decreased H3K4me2 and H4K12ac in F1 mouse oocytes. Representative images of H3K4me2 (**A**), H3K9me2 (**C**), H4K12ac (**E**), and H3K27ac (**G**) in F1 mouse oocytes. Scale bar, 20 μm. The fluorescence intensity of H3K4me2 (**B**), H3K9me2 (**D**), H4K12ac (**F**), and H3K27ac (**H**). The total number of oocytes examined is given in parentheses at the top of each column. ** *p* values of <0.01.

Considering the cadmium concentration that affects ovulation and oocyte maturation (Figure 2), a moderate concentration (3.2 mg/L) of cadmium was used for the following study. Epigenetic modification, particularly histone methylation and histone acetylation, plays important and distinct roles during oocyte maturation [22]. As shown in Figure 5A,B, the relative fluorescence intensity of H3K4me2 in the GV-oocytes from the cadmium group was significantly decreased, compared with the control group (60.66 ± 3.28 vs. 98.81 ± 7.05, *p* < 0.01). But the H3K9me2 has a similar intensity of relative fluorescence between the two groups (Figure 5C,D, 85.12 ± 2.96 vs. 75.60 ± 4.29, *p* > 0.05). Meanwhile, the H4K12ac intensity was lower in the cadmium group than in the control group (Figure 5E,F, 75.46 ± 3.66 vs. 104.70 ± 5.61, *p* < 0.01). There was no significant difference in H3K27ac levels between the two groups. These results indicated that cadmium exposure during pregnancy selectively decreased H3K4me2 and H4K12ac in F1 mouse GV-oocytes.

### 2.6. Cadmium Exposure during Pregnancy Altered DNA Methylation of H19 in F1 Mouse Oocytes

**Figure 6:**
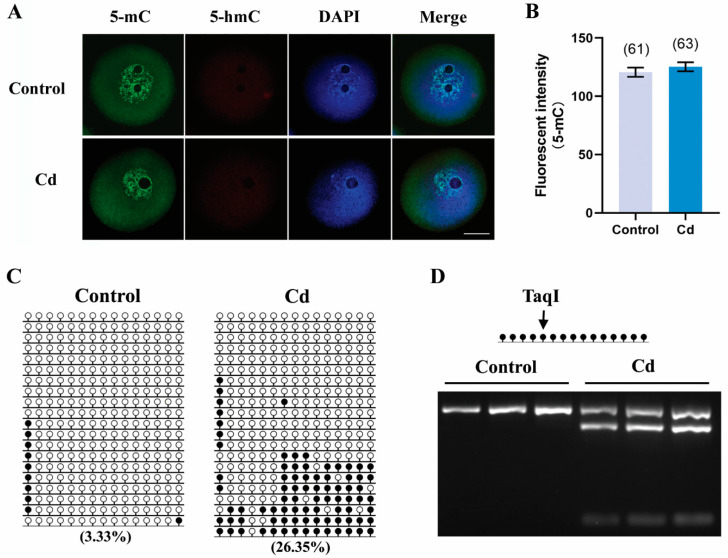
Cadmium exposure during pregnancy altered the DNA methylation of *H19* without affecting the global methylation level in F1 mouse oocytes. (**A**) Representative images of 5-mC (green), 5-hmC (red), and DAPI (blue) in F1 mouse oocytes. Scale bar, 20 μm. (**B**) The fluorescence intensity of 5-mC. The total number of oocytes examined is given in parentheses at the top of each column. (**C**) Methylation profiles of the imprinted *H19* gene were assayed using bisulfite sequencing. Each line represents an individual clone allele. Each circle within the row represents a single CpG site. Black circles and white circles represent methylated and unmethylated CpGs, respectively. The percentage number indicates the DNA methylation level of *H19* in each group. (**D**) Methylation patterns of *H19* detected by COBRA. Restriction enzymes are cleaved only if the recognized TaqI site is methylated. The same bisulfite-treated DNA sample used for sequencing was digested.

DNA methylation, another epigenetic modification, is maintained at a high level in oocytes to ensure normal genome reprogramming after fertilization. If cadmium exposure during pregnancy disturbs global DNA reprogramming, it may cause abnormal DNA methylation of F1 mouse oocytes. To confirm this possibility, 5-mC and 5-hmC in GV-oocytes were examined by immunostaining. As shown in Figure 6A,B, the fluorescent intensity of 5-mC was comparable between the cadmium group and the control group (125.3 ± 3.90 vs. 120.6 ± 3.95, *p* > 0.05). Meanwhile, we did not detect the 5-hmC signals by immunofluorescence staining (Figure 6A). *H19*, a paternally imprinted gene, is a target for environmental insults. Bisulfite sequencing showed that DNA methylation of *H19* in the cadmium group was significantly higher than that in the control group (Figure 6C, 26.35% vs. 3.33%). The COBRA analysis also confirmed that the CpG site recognized by TaqI was partly methylation in the cadmium-treated oocytes, but it was completely demethylation in the control oocytes (Figure 6D). This finding indicated that DNA methylation of *H19* in F1 mouse oocytes was affected by cadmium exposure during pregnancy, but the global methylation can be maintained.

### 2.7. Cadmium Exposure during Pregnancy Caused DNA Damage and Decreased DNA Repair in F1 Mouse Oocytes

**Figure 7:**
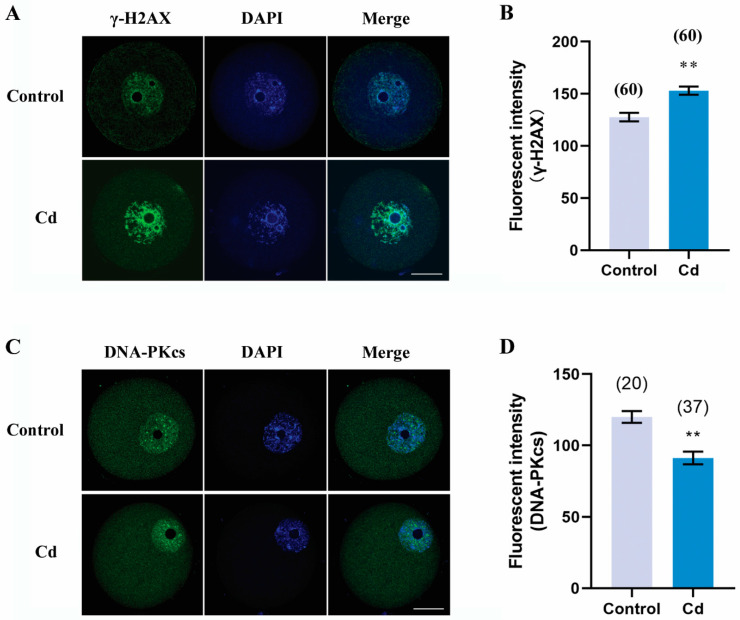
Cadmium exposure during pregnancy caused DNA damage and decreased DNA repair in F1 mouse oocytes. Representative images of γH2AX (**A**) and DNA-PKcs (**C**) in F1 mouse oocytes. Scale bar, 20 μm. The fluorescence intensity of γH2AX (**B**) and DNA-PKcs (**D**). The total number of oocytes examined is given in parentheses at the top of each column (**B**,**D**). ** *p* values of <0.01.

Direct exposure of cadmium to female mice induces DNA damage in oocytes and embryos [6,23]. We then asked whether this mechanism worked on F1 mouse oocytes when cadmium exposure occurred during pregnancy. To confirm this, we used immunofluorescence staining to detect γH2AX as a biomarker for DNA damage. As shown in Figure 7A,B, the fluorescence intensity of γH2AX in F1 oocytes from cadmium-exposed mice during pregnancy was markedly higher than that in the control group (153.00 ± 3.96 vs. 127.60 ± 4.03, *p* < 0.01). Thus, DNA-dependent protein kinase catalytic subunit (DNA-PKcs), as a marker for DNA repair, was also detected. We found that the fluorescence intensity of DNA-PKcs in the cadmium group was significantly decreased compared to the control group (91.28 ± 4.39 vs. 119.90 ± 4.10, *p* < 0.01). Our finding indicated that cadmium exposure during pregnancy caused DNA damage and decreased DNA repair in F1 mouse oocytes.

### 2.8. Cadmium Exposure during Pregnancy Did Not Affect the ROS Level and the Mitochondrial Activity in F1 Mouse Oocytes

**Figure 8:**
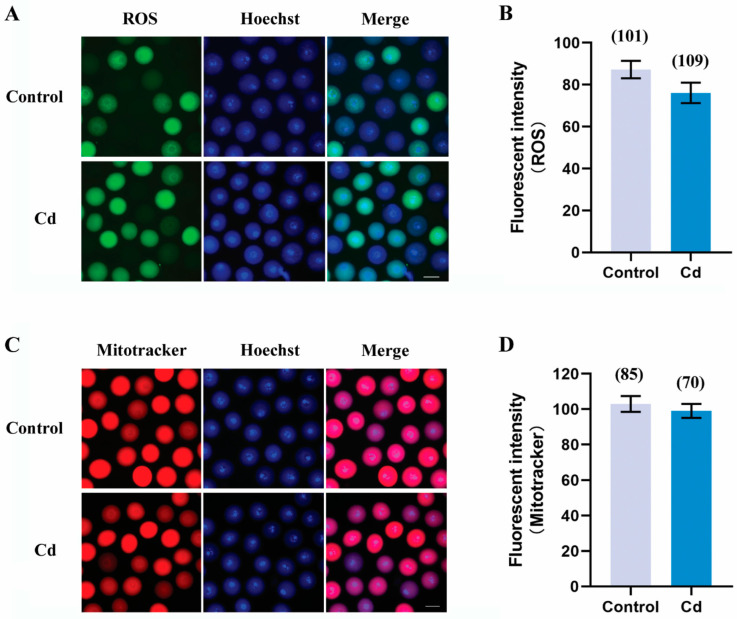
Cadmium exposure during pregnancy did not affect the ROS level and mitochondrial activity in F1 mouse oocytes. Representative images of ROS (**A**) and mitochondria (**C**) in F1 mouse oocytes. Scale bar, 50 μm. The fluorescence intensity of ROS (**B**) and mitochondria (**D**). The total number of oocytes examined is given in parentheses at the top of each column (**B**,**D**).

It is well known that ROS is a source of DNA damage and also participates in the activation of the DNA damage response. Direct exposure of cadmium to female mice increases ROS levels and leads to abnormal mitochondrial distribution and activity in oocytes and embryos [6,23]. We separately used DCFH staining and Mitotracker staining to assess the ROS levels and the mitochondrial activity in oocytes. As shown in Figure 8A,B, the fluorescent intensity of ROS was comparable between the cadmium group and the control group (76.07 ± 4.88 vs. 87.25 ± 4.16, *p* > 0.05). Similarly, there was no significant difference in Mitotracker levels between the two groups (Figure 8C,D, 99.04 ± 3.96 vs. 103.00 ± 4.48, *p* > 0.05). These results indicated that the oxidative stress and the mitochondrial membrane potential in F1 mouse oocytes were normally maintained when the pregnancy mouse was exposed to cadmium.

### 2.9. Cadmium Exposure during Pregnancy Altered the Transcriptome in F1 Mouse Ovaries

**Figure 9:**
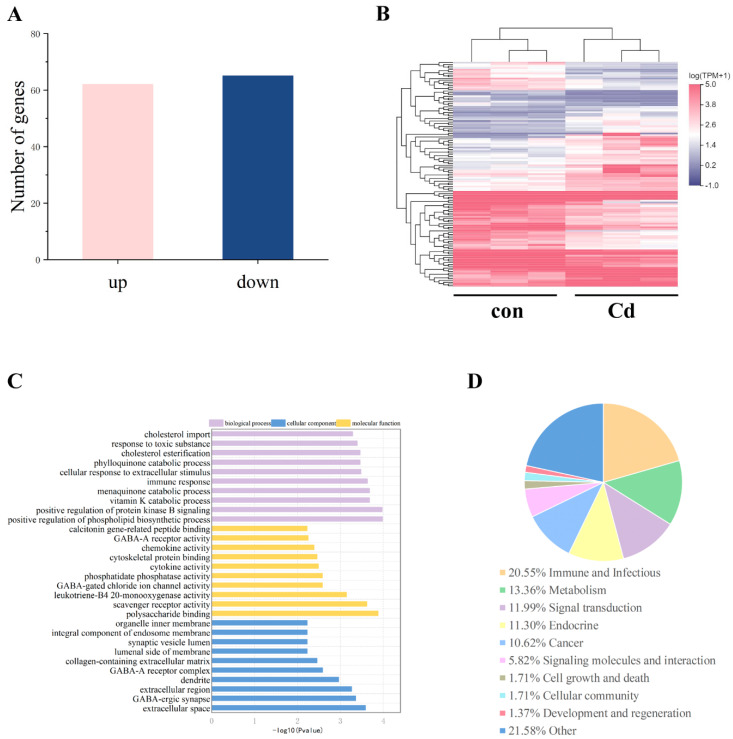
Cadmium exposure during pregnancy affected the transcriptome in the F1 mouse ovary. (**A**) The number of differentially expressed genes (DEGs). (**B**) Heatmap of DEGs in control and cadmium-exposure ovaries. (**C**) GO pathway enrichment analysis for the DEGs. (**D**) KEGG enrichment analysis for the DEGs.

To explore the mechanism by which cadmium exposure during pregnancy affected oocyte maturation and epigenetic modification, we conducted RNA sequencing of F1 mouse ovaries. There were 127 DEGs in the cadmium group compared with the control group (Figure 9A,B). Among the DEGs, 62 were upregulated and 65 were downregulated. GO analysis showed that the DEGs were mainly involved in the phospholipid biosynthetic process, vitamin K catabolic process, protein kinase B signaling, polysaccharide binding, scavenger receptor activity, and extracellular space and region (Figure 9C). KEGG analysis showed that the top four groups of DEGs were involved in immune, metabolism, signal transduction, and endocrine processes (Figure 9D). These findings indicated that cadmium exposure during pregnancy resulted in the disorder of ovarian metabolism and signal transduction in F1 mice.

## 3. Discussion

Cadmium exposure damages the reproduction of both males and females. The mechanism by which cadmium toxicity affects the reproduction of offspring, particularly the germ cell, still needs to be studied. Actually, when the female is exposed to environmental stimuli during pregnancy, the germ cell of the F1 generation is also potentially exposed to the stimuli. While 70–80% of the oocyte population is eliminated by caspase 9-dependent apoptosis during fetal ovarian development and by a caspase 9-independent mechanism during neonatal development [24,25]. If the oocytes were not eliminated after maternal cadmium exposure during pregnancy, the oocyte defects caused by cadmium may be preserved in the offspring oocytes. The current results support the hypothesis that maternal cadmium exposure during pregnancy results in a decrease in the quantity and quality of F1 oocytes and subsequent early embryonic development.

Previous research has shown that maternal cadmium exposure throughout the gestational and lactational period results in a significant decrease in the ovary weight of F1 generation rats [26]. Our research has improved the observations to some degree, as cadmium exposure alone during pregnancy can reduce the ovary coefficient of F1 mice. Although our study did not show abnormalities in the organ coefficients of the liver and kidneys caused by cadmium, the possibility that maternal cadmium exposure interferes with the biochemical functions of the liver and kidney in offspring cannot be excluded. We previously reported that female exposure to cadmium impaired the meiotic maturation of oocytes and subsequent embryonic development [5]. The current results indicated this mechanism worked on F1 mouse oocytes when maternal cadmium exposure occurred during pregnancy. Due to the removal of cadmium exposure after delivery, defects in F1 mouse oocytes can only be induced by cadmium during fetal development. Maternal cadmium exposure during pregnancy impaired the oocyte meiotic maturation both in vivo and in vitro, indicating that cadmium has a weaker impact on the ovarian microenvironment of F1 mice. The failure of early embryonic development after IVF once again indicated that maternal cadmium exposure affects the oocytes of F1 mice. To determine the underlying mechanisms for the impairment of oocyte quality by cadmium, we first investigated the spindle microtubules and chromosome alignment. Consistent with our previous study, several reports have shown that cadmium disrupts spindle assembly checkpoint and chromosomal alignment [5,27,28]. However, our current result revealed that the negative relationship between cadmium and spindle and chromosome had not been maintained in F1 oocytes.

More and more studies have demonstrated that epigenetic modifications are a key functional target for cadmium toxicity [29,30]. However, the understanding of the transgenerational effect of cadmium-induced epigenetic alterations, especially in germ cells, is limited. Our current study investigated epigenetic alterations in F1 oocytes induced by maternal cadmium exposure during pregnancy, including histone methylation, histone acetylation, and DNA methylation. Histone methylation remains relatively stable during the meiotic maturation of oocytes [31]. Dimethylation and trimethylation of histone exhibit different roles during oocyte maturation and early development of the embryo. Research shows that dimethylation of H3K4 and H3K9 is more involved in transgenerational reproductive toxicity than monomethylation and trimethylation [32]. Studies on *C. elegans* have also shown that transgenerational reproductive effects of environmental toxins are associated with H3K4me2 [33]. Our data suggest that the downregulation of H3K4me2 but not H3K9me2 may have partially contributed to the defects in F1 mouse oocytes and the failure of early embryonic development induced by maternal cadmium exposure during pregnancy. In contrast, histone acetylation exhibits higher levels in fully grown GV-oocytes, followed by stage-dependent dynamic changes with the resumption of meiosis, and reaching lower levels in MII-oocytes [31]. We found that maternal cadmium exposure during pregnancy resulted in a decrease in H4K12ac in F1 mouse GV-oocytes. It has been demonstrated that insufficient deacetylation of H4K12ac in oocyte meiotic maturation results in aberrant spindle and chromosome [34]. Although maternal cadmium exposure leads to more F1 oocytes carrying abnormal spindles, it lacks statistical significance. Therefore, the early occurrence of H4K12ac deacetylation in GV-oocytes induced by cadmium does not lead to spindle abnormalities. A previous study focused on H3K27ac in mouse oocytes and early embryos has profiled the hyperacetylation of H3K27 in GV-oocytes [35]. Our result indicated that maternal cadmium exposure during pregnancy did not interfere with the levels of H3K27ac in F1 mouse oocytes. One possible explanation for this result may be the rapid transition of H3K27ac.

During gametogenesis, the genome of primordial germ cells undergoes global demethylation and establishment of sex-specific germ cell methylation patterns, including DNA methylation of imprinted genes. Studies on fish have shown that DNA hypermethylation in female fish is positively correlated with the contamination level of fish, as well as with the oocyte stage during gonadal maturation [36]. Our results indicated that maternal cadmium exposure during pregnancy did not disturb the high levels of 5-mC while increasing the DNA methylation level of the imprinted gene *H19* in F1 GV-oocytes. One probable reason is that cadmium did not interfere with genome-wide reprogramming of DNA methylation in primordial germ cells, but it has an influence on the erasure or reestablishment of imprints, at least in surviving oocytes. Abnormal DNA methylation of *H19* in GV-oocytes is also partially responsible for embryonic development failure induced by cadmium.

We also demonstrated that DNA damage and repair were involved in cadmium toxicity to F1 mouse oocytes. This is consistent with other reports and our previous observation that cadmium induces DNA damage and inhibits gene expression of DNA repair in mouse preimplantation embryos [6]. DNA damage and repair cause epigenetic changes, while DNA methylation and histone modification also promote DNA damage and impact DNA repair efficiency [37]. For instance, all sites of DNA damage showed a reduction in H3K4me2/3, and homologous recombination repair of double-strand breaks impacts regional DNA methylation and histone H3 methylation. Lysine demethylases (KDMs) mediated the demethylation of H3K4me2/3 at DNA damage sites, which is an important step in promoting DNA damage response [38]. Our study confirms that DNA damage and repair involved in cadmium toxicity is linked to the reduction in H3K4me2.

As widely acknowledged, a unified mechanism of cadmium toxicity is that mitochondrial abnormalities and ROS accumulation induced by cadmium lead to DNA damage and ultimately cell death [39]. Mitochondria play a critical role in cellular adaptation to various stressors, including oxidative stress and DNA damage, and also support essential processes during oocyte maturation and early embryonic development [40]. Unexpectedly, our study found that maternal cadmium exposure during pregnancy did not cause abnormalities in ROS and mitochondrial membrane potential within F1 oocytes. One potential explanation is that considering the removal of cadmium exposure after delivery, oocytes with abnormal ROS and mitochondria are eliminated during neonatal development. Another possible explanation is that only oocytes with the ability to restore normal levels of ROS and mitochondria can survive. These results highlight the complex interplay between maternal cadmium exposure, ROS levels, mitochondrial activity, DNA damage, and quality of F1 mice oocytes.

While our research findings shed light on the direct harm of maternal cadmium exposure during pregnancy to offspring oocytes, the alterations in the microenvironment in which the oocytes are located are still unknown. We analyzed the transcriptome of F1 mouse ovaries. The results showed that maternal cadmium exposure elicits modifications in the expression of genes associated with immune response, metabolism, signal transduction, and endocrine regulation. Given the ceasing of genomic transcription in GV-oocytes, the majority of these cadmium-affected genes were identified in ovarian somatic cells. The ovarian microenvironment is decisive for the quality and maturation of oocytes. Hence, alterations in the microenvironment of offspring ovaries, particularly in metabolism and endocrine regulation, also contribute to the disruption of oocyte maturation and failures in early embryonic development caused by cadmium exposure during pregnancy.

In conclusion, our study provides valuable insights into the direct harm of maternal cadmium exposure to offspring oocytes. The underlying mechanisms of cadmium reproductive toxicity can be attributed to abnormal epigenetic modifications, including histone methylation, histone acetylation, and DNA methylation of imprints. These epigenetic changes caused by cadmium exposure during pregnancy may occur in the early stages of oocyte development, which can have a negative impact on early embryonic development. A comprehensive understanding of the influence of maternal cadmium exposure on offspring oocytes is essential for assessing the inter- and multi-generational harm of cadmium exposure and developing strategies to mitigate its adverse effects. Elucidating the specific mechanisms involved and exploring the potential for transgenerational epigenetic inheritance will contribute to a better understanding of the long-term consequences of environmental toxicants on reproductive health. The development of epigenetic-modified drugs or nutrients may have the potential to reduce the transgenerational reproductive toxicity of cadmium.

## 4. Materials and Methods

### 4.1. Chemicals

All chemicals were purchased from Sigma Chemical (St. Louis, MO, USA), unless otherwise stated.

### 4.2. Animals and Experimental Design

All procedures executed in this study were approved by the Animal Care and Use Committee of Yangzhou University. The healthy 8-week-old ICR mice were obtained from the Experimental Animal Center of Yangzhou University. The mice were housed at 24–26 °C, 12 h light-dark cycles, and with free access to standard food and water. The standard food was purchased from Anlimao Company (Yangzhou, China). After two weeks of adaptive feeding, female mice were mated with male mice and checked for pregnancy the next morning, while a vaginal plug was considered to have successfully copulated. The pregnant female mice were randomly divided into four groups and exposed to cadmium during pregnancy. Cadmium exposure and the dosage were determined based on the previous literature and preliminary experiments [5,6]. For the experimental group, the pregnant female mice were given ad libitum access to water containing cadmium chloride at 0.32 mg/L, 3.2 mg/L, and 32 mg/L. The maximum dose used in this experiment (32 mg/L) was 6% of LD_50_ for mice, and much lower than the maximum detectable dose in the environment (75 mg/L) [41,42]. After the F1 mice were born, the mother mice were fed a regular diet and water without the addition of cadmium chloride. The 8-week-old F1 female mice were used for subsequent experiments.

### 4.3. Organ Coefficient, MII-Oocyte Collection, and In Vitro Fertilization (IVF)

F1 female mice were injected intraperitoneally with 10 IU eCG (equine chorionic gonadotrophin) (Ningbo Second Hormone Factory, Cixi, China), followed by injection with 10 IU hCG (human chorionic gonadotrophin) (Ningbo Second Hormone Factory, Cixi, China) 48 h later, and sacrificed 14 h later. Mouse weight and organ weight (ovary, liver, and kidney) were measured. The organ coefficients were calculated by the ratio of organ weight to mouse weight. MII (metaphase II)-oocytes were flushed out from the oviduct and processed for either in vitro fertilization or analysis of meiotic spindles. The oocytes were transferred into HTF medium containing spermatozoa, which were capacitated in HTF medium for 1 h and further incubated for 5 h at 37 °C and 5% CO_2_. After washings, the eggs were cultured in fresh modified KSOMaa medium for up to 5 days.

### 4.4. Analysis of Meiotic Spindles

The collected oocytes were denuded of cumulus cells by treatment with hyaluronidase (300 μg/mL) for 5 min. MII-oocytes were fixed in 4% paraformaldehyde for 30 min at room temperature, and then permeabilized in 0.5% Triton X-100 for 25 min and blocked in PBS containing 1% BSA for 1 h. Oocytes were incubated with anti-α-tubulin-FITC antibody (Abcam, Cambridge, UK) (1:400 diluted with PBS containing 1% BSA) overnight at 4 °C. After 3 washes in PBS, the oocytes were counterstained with DAPI for 10 min. Finally, the oocytes were mounted with Antifade Mounting Medium (Beyotime, Shanghai, China) on histology slides. Fluorescence signals were examined under a confocal laser scanning microscope (TCS SP8, Leica, Wetzlar, Germany).

### 4.5. GV-Oocyte Collection and In Vitro Maturation

F1 female mice were injected intraperitoneally with 10 eCG and sacrificed 48 h later. Fully grown GV (germinal vesicle)-oocytes surrounded by cumulus cells were obtained by puncturing the ovary with needles in the M2 medium. Ten oocytes in a group were cultured in a pre-warmed 20 μL droplet of the M16 medium under mineral oil at 37 °C and 5% CO_2_. After 14 h, the oocytes were denuded of cumulus cells, and the stage of each oocyte was determined under a stereomicroscope. GV-oocytes denuded of cumulus cells without culture were used for subsequent experiments.

### 4.6. Immunofluorescence Staining and Confocal Microscopy Analysis

Immunofluorescence staining was briefly carried out by fixation, permeabilization, blocking, primary antibody incubation, and secondary antibody incubation. Fixation, permeabilization, and blocking were performed as described in the analysis of meiotic spindles. GV-oocytes were independently incubated with anti-H3K4me2 antibodies (Abcam, Cambridge, UK) (1:500), anti-H3K9me2 antibodies (Abcam, Cambridge, UK) (1:500), anti-H4K12ac antibodies (Abcam, Cambridge, UK) (1:200), anti-H3K27ac antibodies (Active Motif, Atlanta, GA, USA) (1:200), anti-γH2AX antibodies (Abcam, Cambridge, UK) (1:200), and anti-DNA PKcs antibodies (Abcam, Cambridge, UK) (1:200) overnight at 4 °C. After 3 washes in PBS extensively, GV-oocytes were incubated with Goat anti-Rabbit Alexa Fluor-488-conjugated IgG (Invitrogen, Carlsbad, CA, USA) (1:500) for 1 h at room temperature. All antibodies were diluted with PBS containing 1% BSA. After 3 washes in PBS, the oocytes were counterstained with DAPI for 10 min. The oocytes were mounted on histology slides and examined under a confocal laser scanning microscope (TCS SP8, Leica, Wetzlar, Germany). The constant scanning setting was used for sample scanning. Fluorescence intensity levels were assessed by Leica microscope software (LAS AF version 2.6.3, Leica, Wetzlar, Germany).

For 5-methylcytosine (5-mC) and 5-hydroxymethylcytosine (5-hmC) staining, the oocytes were treated with 4 mol/L HCl for 10 min at room temperature after permeabilization and then neutralized with 100 mM Tris-HCl for 10 min at room temperature. After blocking in PBS containing 1% BSA for 1 h, the oocytes were incubated with anti-5-mC antibodies (Abcam, Cambridge, UK) (1:200) and anti-5-hmC antibodies (Abcam, Cambridge, UK) (1:200) overnight at 4 °C. After 3 washes, the oocytes were incubated with Goat anti-mouse Alexa Fluor-488-conjugated IgG (Beyotime, Shanghai, China) (1:500) and Goat anti-rabbit TRITC-conjugated IgG (ABclonal, Wuhan, China) (1:500) at room temperature for 1 h. All antibodies were also diluted with PBS containing 1% BSA. Other procedures were performed as described above.

### 4.7. DNA Methylation Analysis

DNA methylation analysis was carried out by bisulfite sequencing and combined bisulfite restriction analysis (COBRA) according to our previous reports [6,43]. DNA was isolated from the GV-oocytes using a DNeasy Blood and Tissue Kit (Qiagen, Dusseldorf, Germany). Bisulfite treatment of DNA was carried out using DNA Methylation-Gold Kit (ZYMO, Orange, CA, USA) according to the instructions of the manufacturer. Bisulfite-treated DNA was used for nested PCR amplification. The first-round primers used for *H19* were 5′-GAGTATTTAGGAGGTATAAGAATT-3′ and 5′-ATCAAAAACTAACATAAACCCCT-3′. An amount of 2 μL of the first-round PCR product was used as a template for the second round. The second-round primers used for *H19* were 5′-GTAAGGAGATTATGTTTATTTTTGG-3′ and 5-CCTCATTAATCCCATAACTAT-3′. The PCR products were gel-purified. The purified fragments were cloned into the pMD19-T Vector (TaKaRa, Shiga, Japan), and the positive clones confirmed by PCR were sequenced. For COBRA, the purified fragments were digested with TaqI restriction enzymes (NEB, Ipswich, MA, USA), and then the digested fragments were electrophoresed on 3% agarose gel.

### 4.8. ROS Measurement and Mitochondria Staining

The ROS levels in GV-oocytes were measured using a ROS Assay Kit (Beyotime, Shanghai, China) according to the instructions of the manufacturer. GV-oocytes were incubated in 10 μM Dichloro-dihydro-fluorescein diacetate (DCFH-DA) and 10 μg/mL Hoechst 33342 at 37 °C for 30 min in the dark. For the mitochondrial staining, GV-oocytes were incubated in 100 nM Mitotracker Orange (Invitrogen, Carlsbad, CA, USA) and 10 μg/mL Hoechst 33342 at 37 °C for 30 min in the dark. After being washed, the oocytes were examined under a fluorescence microscope (Eclipse Ti-E, Nikon, Tokyo, Japan).

### 4.9. RNA-Seq and Bioinformatics Analysis

Total RNA was extracted from the ovary using TRIzol reagent (Invitrogen, Carlsbad, CA, USA) according to the instructions of the manufacturer. The RNA was sent to BGI Genomics Company (Shenzhen, China) for RNA-seq library preparation and sequencing. The differentially expressed mRNAs and genes were selected with 2-fold changes and with statistical significance (*p* value < 0.05) by the Dr. TOM platform of BGI Genomic Company. The selected differentially expressed genes (DEGs) were enriched by Gene Ontology (GO) enrichment and by the Kyoto Encyclopedia of Genes and Genomes (KEGG) pathways.

### 4.10. Statistical Analysis

All the experiments were repeated at least three times. Data are presented as means ± standard errors of the mean (SEM). Statistical differences among pooled results were analyzed using a 2-way analysis of variance (ANOVA) using the SPSS software version 19.0.0 (IBM Corp., Amonk, NY, USA). Statistical differences between groups from separate experiments were analyzed using *t* tests in the SPSS software version 19.0.0. A *p* value of <0.05 was considered statistically significant.

## Data Availability

Data is contained within the article.
